# Acute Corneal Hydrops in Keratoconus Patients with Graves’ Orbitopathy

**DOI:** 10.14744/bej.2021.73645

**Published:** 2021-12-17

**Authors:** Aysun Sanal Dogan, Canan Gurdal, Osman Celikay, Rabiatul Busra Akdan Bilen

**Affiliations:** Department of Ophthalmology, University of Health Sciences, Diskapi Yildirim Beyazit Training and Research Hospital, Ankara, Turkey

**Keywords:** Corneal hydrops, Graves’ orbitopathy, intraocular pressure, keratoconus, thyroid associated orbitopathy

## Abstract

Presented are 2 cases of acute corneal hydrops in keratoconus with Graves’ orbitopathy (GO). Two patients (Case 1: female, 54 years old; Case 2: male, 33 years old) with coexisting keratoconus and GO demonstrated typical findings of acute corneal hydrops (ACH) in 1 eye during the active stage of orbitopathy. There was no history of trauma. The ACH healed with scarring after management with a therapeutic contact lens and medical treatment in each patient within 3 months and 5 months, respectively. The development of ACH in keratoconus patients has previously been reported to be associated with male gender, ethnicity, age, eye rubbing, trauma, rapidly progressive disease, atopy, and vernal conjunctivitis. GO involves ocular surface inflammation and fluctuation of intraocular pressure. Active GO can be a risk factor for ACH in keratoconus patients.

## Introduction

Acute corneal hydrops (ACH), resulting in acute stromal and epithelial edema due to rupture in the descement membrane, occurs in approximately 2.6% of eyes with keratoconus (1). Sudden onset redness, photophobia, pain, and blured vision are major symptoms. Although this self-limiting condition resolves within months, it may lead to serious complications such as low vision, scar development, corneal vascularization, and perforation (1). The association of ACH with keratoconus was shown to be associated with male gender, ethnicity, age, eye rubbing, trauma, atopy, and vernal conjunctivitis (1, 2).

Graves’ orbitopathy (GO) is the most frequent extrathyroidal manifestation of Graves’ disease. It is associated with thyroid diseases as Graves’ disease and Hashimoto’s thyroiditis or without thyroid abnormalities (3). This autoimmune condition ends up with expansion of orbital content secondary to inflammation of orbital tissue, augmented adipogenesis and accumulation of glycosaminoglycans within the extra-ocular muscles (3). GO causes ophthalmic pathologies such as proptosis, diplopia, lid retraction, dry eye, optic neuropathy, exposure keratopathy, increased intraocular pressure (IOP) (3, 4). To the best of our knowledge, there is no reported ACH case in a GO patient with keratoconus.

## Case Report

**Figure 1.**
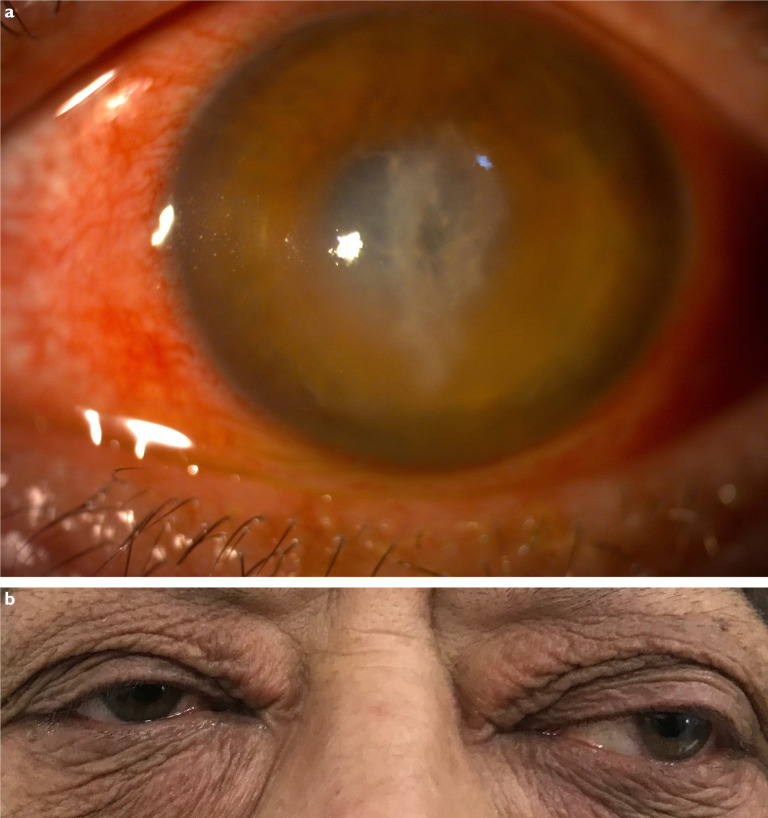
**(a)** Biyomicroscopic image of Case 1, left cornea edema in healing period with scarring. **(b)** Frontal photography of Case 1 demonstrative features of Graves’ orbitopathy.

**Case 1 –** A 54-year-old female patient with a history of Hashimoto thyroiditis and multiple sclerosis that is on levotyroxine sodyum 0.1 mg/day per orally for the past 10 years admitted with blurred vision, pain, and redness in the left eye. She had finger counting from 1 m in the left eye, and conjunctival hyperemia, chemosis, corneal edema (Fig. 1a). There were lid lag, lid retraction, conjunctival chemosis, and proptosis. Corrected IOP were 18.42 OD - 14.24 OS. Hertel measurement was 24 mm OD, 26 mm in OS (Fig. 1b). Keratoconus was detected and ACH was present in the left eye. The hormone levels were thyroid-stimulating hormone (TSH) 0.29 uIU/mL (normally expected levels (EL): 0.34–5.60 uIU/mL), fT4 0.95 ng/dL (EL: 0.58–1.6 ng/dL), fT3 3.18 pg/mL (EL:2.5–3.9 pg/mL).

**Figure 2.**
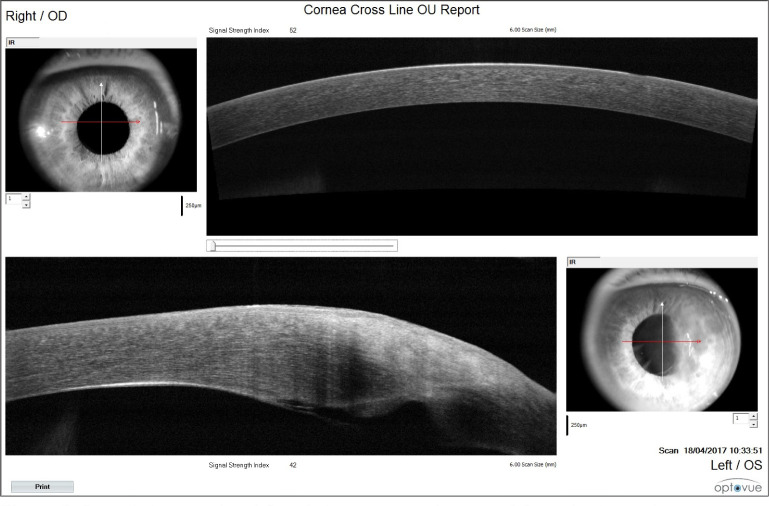
Frontal photography of Case 2 demonstrative features of Graves’ orbitopathy.

**Case 2 –** A 33-year-old male admitted with an abrupt start of redness, epiphora, pain, blurred vision in the left eye. Conjunctival hyperemia, chemosis, and ACH in the left eye was detected. Corrected IOP were 21.93 OD - 21.32 OS. He had diagnoses of keratoconus and Graves diseases. Proptosis was detected by Hertel exophthalmometer measurements of 26 mm OD, 27 mm OS (Fig. 2). The hormone levels were TSH 0.003 uIU/mL (EL: 0.34–5.60 uIU/mL), fT4 1.68 ng/dL (EL: 0.58–1.6 ng/dL), fT3 5.02 pg/mL (EL: 2.5–3.9 pg/mL).

**Figure 3.**
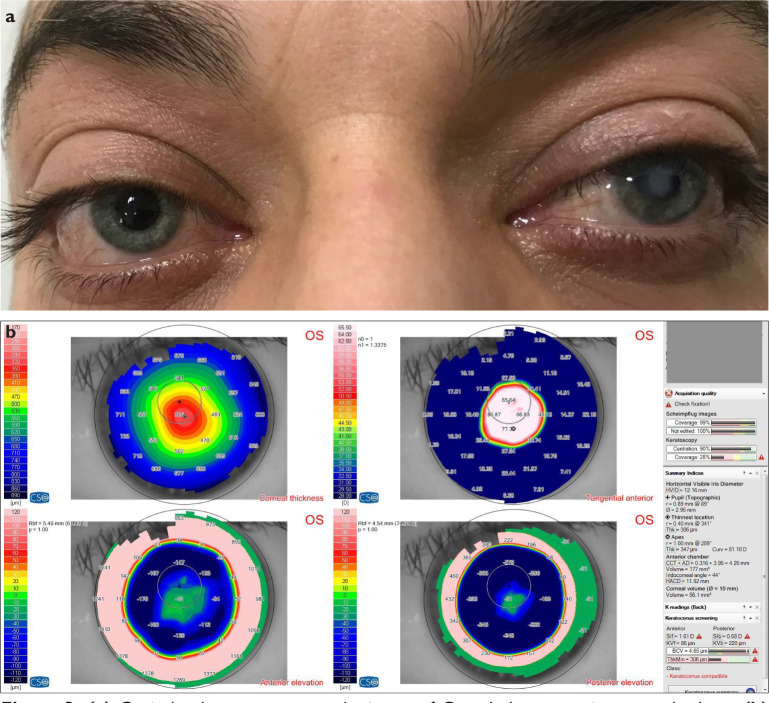
**(a)** Optical coherence tomography image of Case 1 demonstrative corneal edema. **(b)** Corneal topography of Case 2.

None of the cases had positive history for trauma, ocular surgery (including corneal cross-linking) or familial keratoconus. Their keratoconus and ACH were proven with anterior segment imaging: anterior segment optical coherence tomography (OCT, RTVue-XR, Optovue Inc., Fremont, CA) (Figs. 3a and b) and corneal topography (Sirius; Costruzione Strumenti Oftalmici, Florence, Italy).

For the both cases, the diagnoses were, GO, keratoconus and ACH. Therapeutic contact lens was applied, topical medical treatment of cyclopentolate 1% 3 × 1/day, loteprednol 0.5% 5 × 1/day, preservative-free lubricant 6 × 1/day, hypertonic saline %5 3 × 1/day was administered. In the follow-up, both corneas were healed with corneal scars in 3 and 5 months, respectively.

## Discussion

Keratoconus patients have typical corneal biomechanical changes those were thought to be triggered by environmental and endogenic factors such as genes, hormones, atopy, eye rubbing, contact lens wear, and some have comorbid diseases (5). There are controversial reports about association between autoimmune diseases and keratoconus (5). Thyroid gland dysfunction prevalence among patients with keratoconus is 13.6% (6). Our cases had coexistence of keratoconus as well as Hashimoto thyroiditis and Graves’ disease, respectively. It was noteworthy that both patients had ACH which is very rare condition even in normal keratoconus cases (1).

In keratoconus, thin corneal section is susceptible to distending IOP that leads to cone formation (7). IOP distending forces exceeding corneal resistance may lead to the descement membrane rupture endup with ACH (1, 7). Atopy, allergic eye diseases, trauma, eye rubbing, and age are previously reported as the risk factors for the development of ACH (2). Our patients had none of these mentioned risk factors. Moreoever, both patients were older than preponderance age, that is around 25 years old (1). Their ages were clues that they were free of active progression stage. Cornea cross-linking is a procedure which stops the progression of the keratoconus (8). Both of the patients had negative history for cornea cross-linking aplication.

Howard et al. (9) report a 12 years old case with down syndrome, keratokonus, hyperthyroidism and developed ACH, but they do not mention if the child had GO.

We have two hypothesis to explain the co-incidence of ACH with keratoconus in GO. Although keratoconus is first described as a non-inflammatory pathology, in recent studies, increased inflammatory molecules that are thought to contribute to corneal damage in the cornea and tear film layer have been detected (10). Allergic eye diseases those were consequence of inflammatory process are one of the known etiological factors for development of ACH in keratoconus. Therefore, it is our first hypothesis that ocular surface inflammation that is detected in GO may be attributed as an additional etiological factor (11).

In GO, possible mechanisms reported for IOP elevation include increased episcleral venous pressure, contracted extraocular muscles, especially compression of the globe in eye movements leads transient IOP peaks, and trabecular damage through mucopolysaccharide deposition (3, 4). Our second hypothesis for explanation of ACH in keratoconus is IOP increases which are seen during the disease course in GO patients (4). McMonnies et al. (7) demonstrated that even the light digital pressure through adnexial skin doubles the baseline IOP.

## Conclusion

GO can be described as a risk factor for ACH in keratoconus patients. Keratoconus patients with GO must be followed up closely for this visually significant complication.

### Disclosures

**Informed consent:** Written informed consent was obtained from the patient for the publication of the case report and the accompanying images.

**Peer-review:** Externally peer-reviewed.

**Conflict of Interest:** None declared.

**Authorship Contributions:** Involved in design and conduct of the study (ASD, CG); preparation and review of the study (ASD, CG, OC, RBAB); data collection (ASD).
